# Type 2B von Willebrand disease with or without large multimers: A distinction of the two sides of the disorder is long overdue

**DOI:** 10.1371/journal.pone.0179566

**Published:** 2017-06-22

**Authors:** Alessandra Casonato, Viviana Daidone, Eva Galletta, Antonella Bertomoro

**Affiliations:** 1 Thrombohaemorrhagic Disorders Unit, Department of Medicine, University of Padua, Padua, Italy; 2 Internal Medicine, Department of Medicine, University of Padua, Italy; Institut d'Investigacions Biomediques de Barcelona, SPAIN

## Abstract

Most, but not all patients with type 2B von Willebrand disease (VWD)—which features gain-of-function mutations in the A1 domain of von Willebrand factor (VWF)—have no circulating large VWF multimers. Similarities and differences were analysed in 33 type 2B patients, 12 with a normal and 21 with an abnormal multimer pattern, to see whether they should be considered separately. The minimum aggregating dose of ristocetin was similarly reduced in both patient groups, and modulated by their underlying VWF mutations. Platelet VWF content was normal in all patients lacking in large multimers, but sometimes reduced in those with a normal multimer pattern. All the former patients and none of the latter had persistent or transient thrombocytopenia. A short VWF half-life (affecting plasma VWF levels) was seen in both groups, but more pronounced in patients without large multimers. Bleeding scores were also high in all patients, but more so in those without large multimers, apparently regardless of their platelet count. The marked phenotypic heterogeneity of type 2B VWD concerns not only patients’ VWF multimer pattern, but also their bleeding risk, and consequently their appropriate treatment too. Hence the need to clearly distinguish between type 2B VWD with normal or abnormal VWF multimers.

## Introduction

Type 2B von Willebrand disease (VWD) is characterised by a greater affinity of von Willebrand factor (VWF) for platelet glycoprotein Ib (GPIb) [1, 2]. *In vivo*, the defect induces the spontaneous binding of type 2B VWF to platelets without any contribution of endothelial cell injury or high shear stress, and with a consequent loss of large VWF multimers [3]. *In vitro*, the defect is revealed by an enhanced ristocetin-induced platelet aggregation (RIPA) [4,5], expressed as the capacity of a patient’s platelet-rich plasma (PRP) to aggregate at lower than normal ristocetin concentrations [1, 4–6]. Gain-of-function mutations in exon 28 of the *VWF* gene, encoding for the A1 domain of VWF, are responsible for type 2B VWD [7, 8].

VWF has a polymeric structure consisting of oligomers of the same subunit that range in size from 400,000 to more than 10 million Daltons [9,10]. The largest VWF multimers have the greatest haemostatic capacity, i.e. they are better able to bind subendothelial matrix or platelets [11]. In the classic form of type 2B VWD, mutated VWF is synthesised and assembled normally (so it occurs normally in platelets and endothelial cells), but—after its release–the large multimers are removed due to their spontaneous binding to platelets. ADAMTS13 contributes to this process because the type 2B VWF bound to platelets becomes sensitive to proteolysis by ADAMTS13 [12]. The resulting loss of large VWF multimers in patients with type 2B VWD is associated with moderate or severe, persistent or transient thrombocytopenia, sometimes associated with the presence of giant platelets and circulating platelet aggregates [13,14]. Thrombocytopenia may become apparent, or be exacerbated, as a result of pregnancy [15,16], surgery [17], or DDAVP (1-desamino-8-D-arginine vasopressin) administration [18,19]. Thrombocytopenia is believed to contribute to the onset of the severe bleeding tendency of patients with type 2B VWD [13].

VWF may have a greater affinity for platelets even in the presence of a normal multimer pattern in 2B VWD, however, as seen in variants such as type New York [20], type Malmo, and others [21]. These conditions differ from classic type 2B VWD not only in the associated VWF multimer pattern, but also in that patients never suffer from thrombocytopenia. The aim of the present study was to compare the main features of type 2B patients with and without large VWF multimers to establish whether they should be considered separately.

## Materials and methods

Patients and healthy controls (matched for age and sex) were studied in accordance with the Helsinki Declaration, after obtaining their written informed consent (from the parents or the guardians for the minor subjects). The study was approved by our institutional review board (Ethics Committee of University of Padua and Padua Hospital; approval number 730Pt).

Type 2B VWD patients were enrolled for the present study when they attended their annual check-up at our Haemostatic Center.

### Haemostatic tests

Blood samples were drawn from the antecubital vein and anticoagulated using 3.8% sodium citrate (1/10 vol/vol), which was supplemented with 60 mM N-ethylmaleimide (NEM), 50 mM EDTA, and with 200 kallikrein inhibitory units (KIU)/mL of aprotinin when blood samples were collected to assess platelet VWF content. Basic haemostatic analyses, i.e. plasma VWF antigen (VWF:Ag), VWF collagen binding (VWF:CB), VWF ristocetin cofactor (VWF:RCo), ristocetin-induced platelet aggregation (RIPA), VWF multimers and FVIII, were conducted as described elsewhere [22]. Platelet VWF content was measured by washing PRP with the addition of 3% EDTA in PBS buffer three times, as described previously [23]. DDAVP (Emosint, Sclavo, Italy) was administered subcutaneously at a dose of 0.3 μg/kg, and blood samples were collected before and then 15, 30, 60, 120, 180, 240, 360 and 480 min, and 24 hours afterwards. The time courses of the VWF:Ag, VWF:CB and FVIII plasma concentrations after DDAVP were analysed using a one-compartment model with first-order input and output kinetics, as previously reported [24].

VWF propeptide (VWFpp) was analysed with a home-made ELISA method that uses CLB-Pro 35 as the first antibody and CLB-Pro 14.3 HPR-conjugated as the second (Sanquin, Amsterdam, Netherlands).

### Genetic analysis

Genomic DNA was extracted from peripheral blood leucocytes using the QIAamp DNA blood Mini Kit (QIAGEN, Hilden, Germany). Exon 28 of *VWF* gene was amplified and sequenced using primers chosen according to the *VWF* sequence identified by Mancuso et al [25].

### Statistical analysis

Laboratory data were expressed as mean ± standard error of the mean (SEM). The unpaired t-test was used to compare all the results and Pearson’s correlation analysis was conducted to assess the association between the parameters. Welch’s correction was used when variances were not equal. P values below 0.05 were considered statistically significant.

## Results

### Patients

Thirty-three patients with type 2B VWD from 14 unrelated families were studied: 21 patients (from 8 families; 13 males and 8 females; ranging between 1 and 81 years of age) had an abnormal VWF multimer pattern (lacking in large multimers); and 12 patients (from 6 families; 5 males and 7 females; ranging between 11 and 76 years of age) had a normal VWF multimer pattern.

### Haemostatic pattern

**Fig 1:**
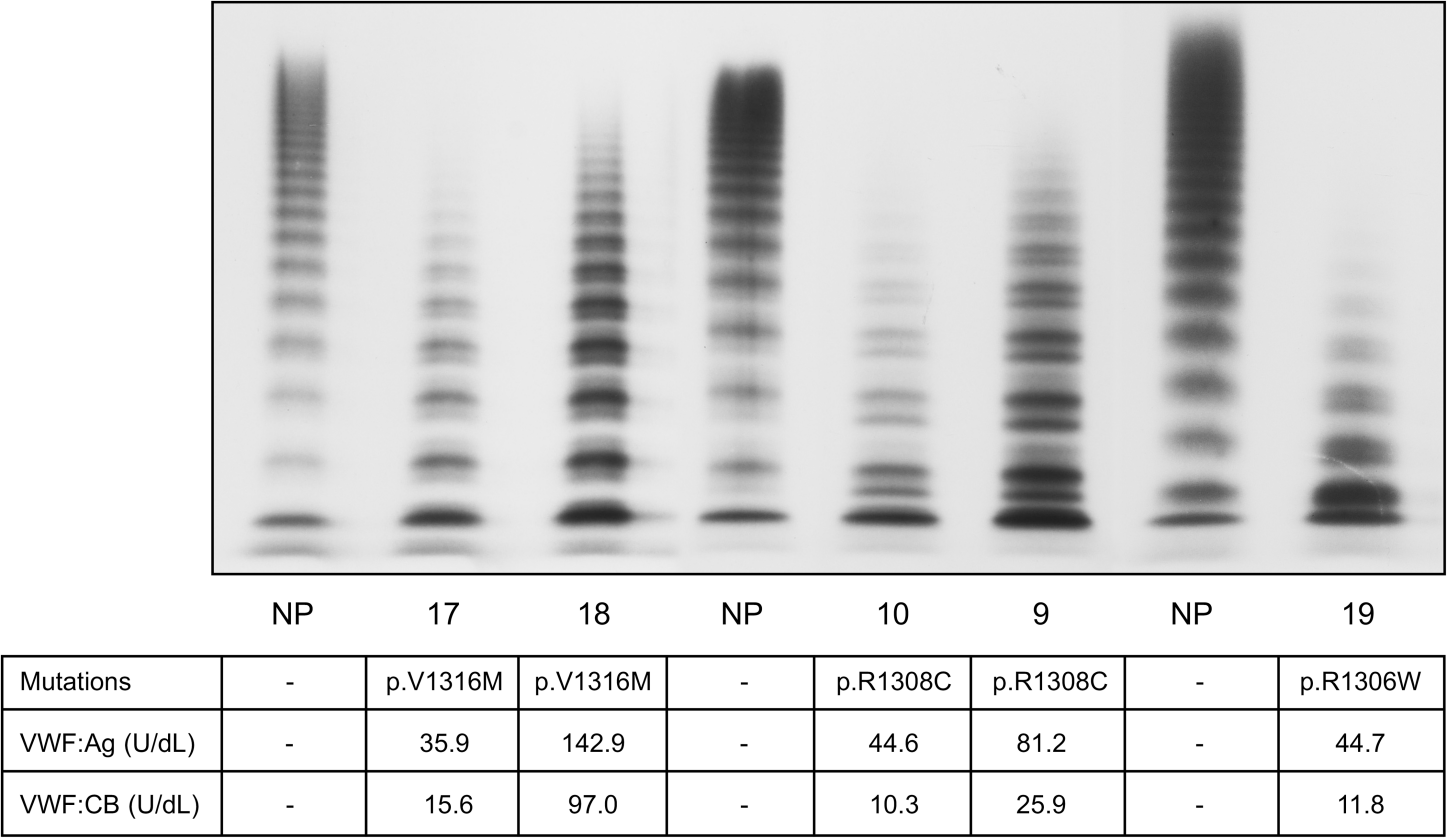
Plasma VWF multimer pattern obtained in 1.8% agarose gel using ^125^I-labelled anti-VWF antibody in type 2B VWD patients lacking in large VWF multimers. Patients are identified by the numbers entered in Table 1.

Tables 1 and 2 show the patients’ main haemostatic parameters. The patients lacking in large VWF multimers were found to carry the mutations p.R1308C (the most common in our cohort), p.R1306W and p.V1316M. These patients were characterised by a significantly reduced VWF:RCo/VWF:Ag ratio (VWF:RCo ratio) (mean 0.50+/-0.13 vs normal ≥ 0.75), and VWF:CB/VWF:Ag ratio (VWF:CB ratio) (0.28+/-0.16 vs normal ≥0.75), consistent with their lack of large VWF multimers (Fig 1). Their minimum aggregating dose of ristocetin (MADR) ranged from 0.15 to 0.75 mg/mL (normal ≥ 1.0 mg/mL), the lowest MADR being found in patients carrying the p.V1316M mutation (0.15 mg/mL), and the highest in patients carrying the p.R1308C mutation. Some of the patients lacking in large VWF multimers (9 cases) also showed spontaneous platelet aggregation (SPA), with values ranging between 7% and 22.5%, whereas no SPA was ever seen in healthy controls (Table 1). Platelet VWF antigen was always normal in the type 2B VWD patients who were lacking in large VWF multimers (mean 125.9 +/-32.1 U/dL vs normal range 70–140 U/dL), while these patients had significantly lower levels of the intermediate multimers, an accumulation of small multimers, and a stronger representation of the satellite bands of each oligomer (Fig 1). No differences in large VWF multimer representation came to light in relation to the three mutations identified in the patients considered (Fig 1). On the other hand, the severity of their shortage of large multimers seemed to be influenced by patients’ VWF:Ag levels: those with the highest VWF levels under basal conditions (patients 9 and 18, Fig 1) had the least pronounced reduction in large multimers. Patients’ bleeding tendency was assessed with the Bleeding Assessment Tool (BAT), and found significantly higher than normal. Their bleeding scores (BS) averaged 11.3+/-5.4 and were higher in females (12.3+/-4.1) than in males (10.7+/-6.2); the BS in our healthy controls were in the range of 0–5 in females, and 0–3 in males.

**Table 1:** Main haemostatic findings of the type 2B VWD patients studied showing abnormal VWF multimer pattern.

Patients	Family	Sex/Age	Blood Group	Platelet count x10^3^/μl	PFA 100 sec	*SPA %	§MADR mg/mL	VWF:Ag U/dL	VWF:CB U/dL	VWF:CB ratio	VWF:RCo U/dL	VWF:RCo ratio	Platelet VWF U/dL	FVIII U/dL	BAT F/M	VWF mutations
1	A	F/3	B	290	>264	NA	NA	41.0	4.4	0.11	13.2	0.33	76.4	36.1	NA	p.R1308C
2	A	M/78	O	119	>300	7	0.75	44.7	11.8	0.27	28.4	0.63	115	48.2	1	p.R1308C
3	A	F/38	A	207	>300	0	0.45	67.9	3.6	0.06	33.1	0.48	128	54.5	10	p.R1308C
4	A	M/43	A	163	>300	0	0.6	42.8	10.6	0.25	25.2	0.58	132.7	47.1	10	p.R1308C
5	B	M/26	O	176	>300	8	0.6	17.6	9.2	0.52	10.4	0.59	76.6	38.8	8	p.R1308C
6	B	M/15	A	180	>300	0	0.6	30.9	12.5	0.41	11.4	0.37	79.1	49.3	4	p.R1308C
7	B	M/81	A	165	>300	NA	0.6	166.4	30.0	0.18	112.0	0.67	NA	136.0	NA	p.R1308C
8	B	F/49	A	178	>300	9.5	0.75	23.0	4.2	0.19	13.8	0.63	74.0	50.0	12	p.R1308C
9	B	M/71	O	56	>300	22	0.3	81.2	25.9	0.32	33.75	0.41	146.2	52	NA	p.R1308C
10	C	F/70	O	168	>300	15	0.75	44.6	10.3	0.23	21.4	0.47	202.0	53.2	15	p.R1308C
11	C	M/80	O	182	>300	0	0.45	39.5	9.3	0.24	23.3	0.59	113.90	52.1	14	p.R1308C
12	C	F/60	O	154	>300	NA	0.75	44.6	8.1	0.18	23.0	0.51	127	68.0	18	p.R1308C
13	C	M/37	O	132	>300	0	0.6	51.9	10.6	0.20	25.2	0.48	133.4	34.5	17	p.R1308C
14	C	M/39	O	210	>300	0	0.6	61.3	10.4	0.17	18.5	0.3	119	40.0	6	p.R1308C
15	C	F/14	O	126	>300	0	0.45	35.0	4.0	0.11	12.7	0.38	141	46.0	6	p.R1308C
16	D	M/67	O	60	>300	20	0.15	34.3	10.5	0.31	14.0	0.41	142.50	43.5	20	p.V1316M
17	E	F/24	O	65	>300	22.5	0.15	35.9	15.6	0.44	14.4	0.40	140.3	35.9	13	p.V1316M
18	E	F/1	O	28	>300	NA	NA	142.9	97.0	0.68	102.7	0.72	135	98.1	NA	p.V1316M
19	F	M/44	O	116	>300	15.7	0.3	44.7	11.8	0.48	11.4	0.71	130	27.7	10	p.R1306W
20	G	M/58	B	213	>300	12.6	0.3	48.4	4.6	0.10	13.5	0.28	175	56.4	17	p.R1308C
21	H	M/40	A	158	>300	NA	0.45	32.5	14.7	0.45	18.7	0.59	130	39.0	NA	p.R1306W
Normal range				150–350	94–193	0	≥1.0	60–160	65–150	≥0.75	60–130	≥0.75	70–140	60–160	0-5/0-3	

*Spontaneous platelet aggregation

§Minimal aggregating dose ristocetin

VWF:CB/VWF:Ag ratio

VWF:RCo/VWF:Ag ratio

NA: not assessed

**Table 2:** Main haemostatic pattern of the type 2B VWD patients studied characterized by normal VWF multimer pattern.

Patients	Family	Sex/Age	Blood Group	Platelet count x10^3^/μl	PFA100 sec	*SPA %	§MADR mg/mL	VWF:Ag U/dL	VWF:CB U/dL	VWF:CB ratio	VWF:RCo U/dL	VWF:RCo ratio	Platelet VWF U/dL	FVIII U/dL	BAT (F/M)	VWF mutations
1	A	F/48	A	232	NA	12	0.45	160.6	135.6	0.84	132.8	0.83	92.0	78.0	6	p.I1372S
2	A	F/75	A	322	NA	10	0.6	133	144.2	1.1	106	0.79	129.5	126	3	p.I1372S
3	B	M/45	O	204	>300	0	0.45	23.4	21.1	0.9	17.2	0.74	42.6	60.5	20	p.P1266L/ p.C2362F
4	C	F/76	O	236	>300	8.4	0.75	35.4	37.3	1.05	25.4	0.72	66.8	32.0	16	p.R1379C
5	C	F/50	O	211	210	10	0.6	40.6	42.8	1.1	NA	NA	41.2	62.8	8	p.R1379C
6	C	M/53	O	193	199	5.2	0.75	47.1	44.6	0.94	38.0	0.80	47.4	43.8	0	p.R1379C
7	C	F/21	O	293	>300	6	0.6	26.7	27.2	1.01	24.8	0.92	59.1	40.9	6	p.R1379C
8	D	M/49	A	252	175	0	0.6	54.3	55.1	1.01	51.6	0.94	60.3	62.2	NA	p.P1266Q/ p.R1379C
9	D	M/18	A	231	95	0	0.75	151.1	175.5	1.16	153.5	0.87	95.2	148.2	NA	p.P1266Q
10	D	F/24	AB	231	223	6	0.75	46.0	49.1	1.07	40.5	0.83	55.6	64.8	NA	p.R1379C
11	E	M/11	O	220	>300	11.3	0.3	28.8	28.4	0.98	23.0	0.80	54.1	51.5	5	p.R1341W
12	F	F/50	A	204	91	10	0.45	108	101.9	0.94	97.7	0.91	136.4	107.5	16	p.P1266L
Normal range				150–350	94–193	0	≥1.0	60–160	65–150	≥0.75	60–130	≥0.75	70–140	60–160	0-5/0-3	

*Spontaneous platelet aggregation

§Minimal aggregating dose ristocetin

VWF:CB/VWF:Ag ratio

VWF:RCo/VWF:Ag ratio

NA: not assessed.

**Fig 2:**
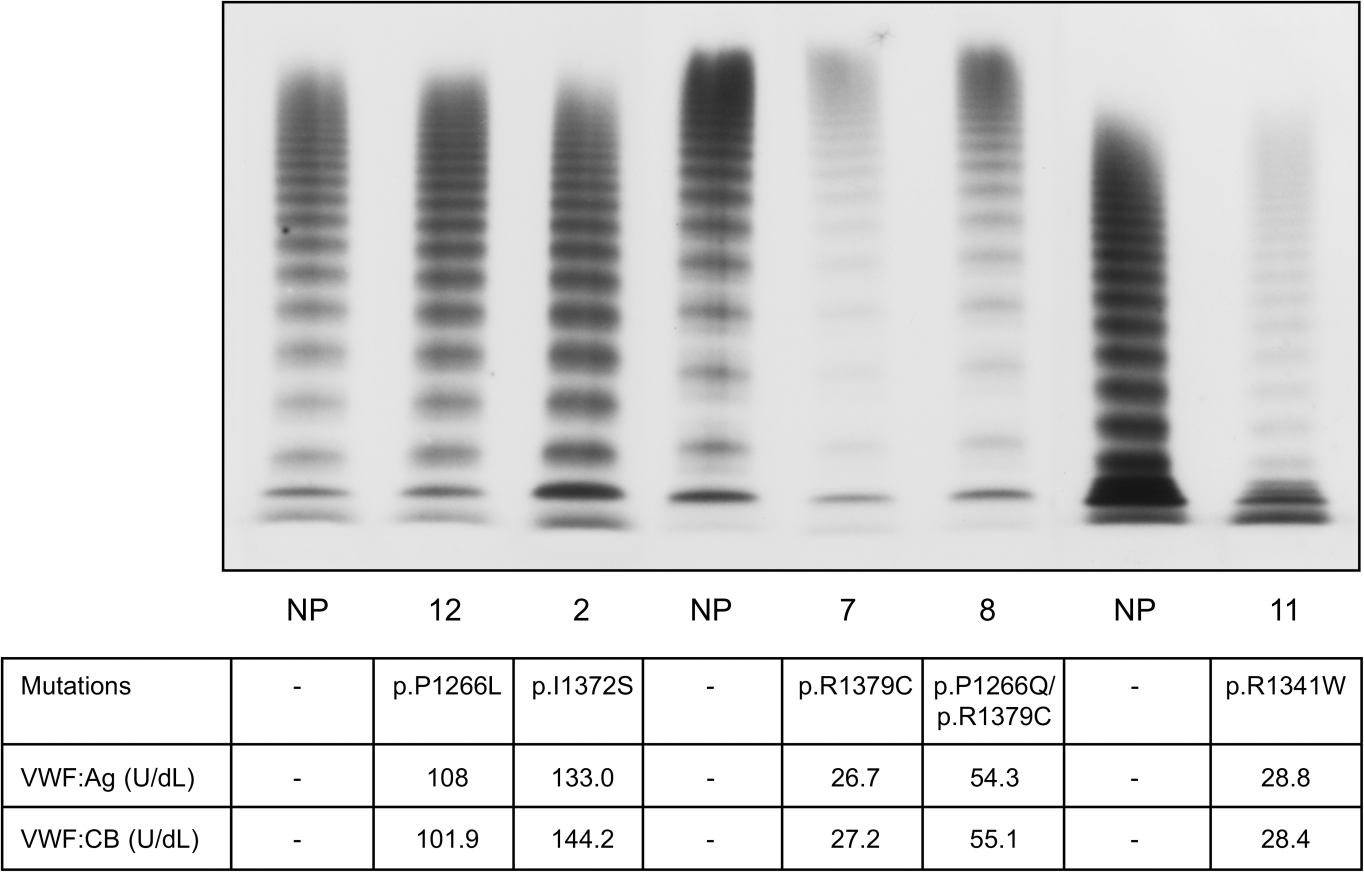
Plasma VWF multimer pattern obtained in 1.8% agarose gel using ^125^I-labelled anti-VWF antibody in type 2B VWD patients with a normal VWF multimer pattern. Patients are identified by the numbers entered in Table 2.

Patients with type 2B VWD who had a normal VWF multimer pattern were found to carry the following mutations: p.I1372S, p.P1266L, p.P1266Q, p.R1379C and p.R1341W. Those carrying the first three of these mutations (p.I1372S, the p.P1266L and p.P1266Q) had normal plasma and platelet VWF levels (Table 2), while those carrying the p.R1379C and p.R1341W mutations had lower plasma and platelet VWF levels, always with normal VWF:RCo and VWF:CB ratios (Table 2, Fig 2). These latter patients had a significantly lower MADR, ranging from 0.3 to 0.75 mg/mL, the most severe reduction being identified in the patient carrying the p.R1341W mutation. Some of the type 2B VWD patients with a normal VWF multimer pattern (9 cases) also showed SPA, with values ranging between 6% and 12% (Table 2). Their BS was higher than normal (8.9+/-6.8) and slightly higher in females (9.2+/-5.5) than in males (8.3+/-10.4). The BS of these patients with a normal VWF multimer pattern varied considerably, however: some had a normal BS, while others (those carrying the p.P1266L mutation, alone or in combination with the p.C2362F mutation) had significantly higher scores (Table 2). Among the type 2B VWD patients lacking in large VWF multimers, on the other hand, all but one had a similarly increased BS (Table 1).

### Platelet count under baseline conditions and after stress

Eight of the 21 type 2B patients lacking in large VWF multimers had low platelet counts under basal conditions, the lowest (51.0+/-20.1 x 10^3^/μL) being identified in the three patients carrying the p.V1316M mutation, who also had giant platelets and persistent thrombocytopenia. No statistical correlation emerged between patients’ BS and their platelet counts (p = 0.7), suggesting that their tendency to bleed was not influenced by the number of their platelets. Three patients experienced a significant drop in their platelet count during pregnancy, which fell by about 60% immediately before delivery, whatever its initial level (mean 187+/-55.7 x 10^3^/μL at the baseline, and 77.8+/-45.7 x 10^3^/μL at delivery). Another patient, who developed heart failure, hypertension and Alzheimer’s disease in old age, showed a significant and persistent drop in platelet count (40 x 10^3^/μL as opposed to 119 x 10^3^/μL twenty years earlier) associated with an increase in VWF:Ag from 43 U/dL 81.2 U/dL. This inverse relationship between platelet counts and VWF levels was particularly evident in patients administered DDAVP because of the short time it took for the increase in VWF to develop. These patients experienced a mean drop in their platelet count from 160.8+/-50.8 x 10^3^μL before to 21.6+/-10.3 x 10^3^μL thirty minutes after DDAVP was administered subcutaneously. Within 360 minutes, their platelet count recovered to near its pre-DDAVP value, however (120+/-51.8 x 10^3^μL), while their VWF:Ag levels rose from 38.4+/-13.6 U/dL at the baseline to 99.2+/-25.7 U/dL thirty minutes after DDAVP, and then fell back to 75.5+/-35.5 U/dL at 360 minutes.

All the patients with type 2B VWD and a normal VWF multimer pattern had normal platelet counts, ranging from 193 x 10^3^μL to 322 x 10^3^/μL under basal conditions (Table 2), and they developed no thrombocytopenia at any time after DDAVP was administered [26].

### Type 2B VWF survival

The results of DDAVP tests and VWFpp measurements were analysed to see how different multimer patterns and associated mutations influenced VWF survival in our type 2B VWD patients. After DDAVP had been administered, the mean VWF:Ag T_1/2_elimination (T_1/2_el) was 4.47h+/-1.16h in patients lacking in large multimers, and 7.34h+/-3.61h in those with a normal multimer pattern, i.e. it was shorter than normal (14.00h+/-6.91h) in both groups of patients. Large VWF multimer survival, explored with the VWF:CB assay, was 3.65h+/-2.11h for patients lacking in large multimers, and 5.934h+/-2.01h for those with a normal VWF multimer pattern (as opposed to 10.6h+/-3.9h in healthy controls).

The mean VWFpp ratio was significantly higher in type 2B VWD patients with abnormal (p<0.0001) or normal (p<0.01) VWF multimer patterns (2.43+/-0.47 and 1.53+/-0.45, respectively) than in healthy controls (1.01+/-0.24). The type 2B patients with normal VWF multimers differed, however, depending on their plasma and platelet VWF levels: those with normal plasma and platelet VWF levels had a mean VWFpp ratio of 0.976+/-0.21, while those with low plasma and platelet VWF levels had a VWFpp ratio of 1.81+/-0.17. Here again, there was evidence of some heterogeneity among the type 2B patients with a normal VWF multimer pattern, though they generally had a less pronounced increase in their VWFpp ratio, consistent with a less marked reduction in their VWF half-life.

## Discussion

Patients whose VWF has a greater affinity for platelet GPIb are identified as cases of type 2B VWD. Although type 2B is characterised in its classic form by the absence of large VWF multimers [1], some type 2B patients have a normal VWF multimer pattern. The first such case was reported by Weiss et al [20] in 1986 and labelled as type New York VWD. Since then, there have been numerous other reports of patients with such a VWD pattern associated with various *VWF* gene mutations. In the present study, we compare these two phenotypes of type 2B VWD (with and without circulating large VWF multimers) to see whether they should be considered together, or clearly distinguished as variants of the same defect. We also discuss the clinical implications of the differences between the two phenotypes.

All the patients with type 2B VWD in our sample, with and without large VWF multimers, revealed a stronger platelet response to VWF in the presence of ristocetin, and much the same minimum ristocetin concentration was capable of inducing platelet aggregation. Some differences emerged in relation to the mutation they were carrying, the lowest MADR values being seen in patients with the p.V1316M mutation (among those lacking in large multimers), and with the p.R1341W mutation (among those with a normal VWF multimer pattern). Both groups of patients included some cases of SPA (not seen in normal subjects), and this phenomenon was also related to the underlying VWF mutation, being most pronounced in association with the p.V1316M and p.R1341W mutations. That all type 2B VWD patients share a VWF with a greater affinity for platelet GPIb is supported by the recent observation that all mutations associated with type 2B VWD are characterised by a higher than normal state of VWF activation (when the VWF A1 domain is in its platelet-binding conformation), as explored with the aid of the AU/VWFa-11 nanobody [27, 28]. Here again, the underlying mutation modulates this phenomenon, since the p.V1316M and p.R1341W mutations were associated with the highest VWF activation state [27]. It seems that type 2B VWD patients with a normal or abnormal VWF multimer pattern are indistinguishable in terms of the affinity of their VWF for platelets, given that the parameters considered here to explore this affinity indirectly (SPA, MADR and VWF activation state) were much the same in the two groups of patients. In some type 2B VWD patients, VWF binding to platelet GPIb may give the factor a steric conformation that makes it susceptible to the action of ADAMTS13, with a consequent proteolytic consumption of large VWF multimers.

Unlike another report in the literature [13], we found no differences in the extent of large VWF multimer loss in relation to a given underlying mutation: the lack of large VWF multimers and the reduction in intermediate oligomers was similar in all the patients with type 2B lacking large multimers, whatever their VWF mutations. There were some differences as concerns patients’ VWF levels, since a less pronounced shortage of large VWF multimers was seen in patients with higher VWF levels under basal conditions. Again in contrast with previous publication [13], all of our patients lacking in large VWF multimers had a lower than normal VWF:RCo ratio, and an even more markedly reduced VWF:CB ratio, whereas our type 2B patients with a normal multimer pattern all had VWF:RCo and VWF:CB ratios similar to those of healthy controls.

What happens after type 2B VWF has interacted with platelets seems to depend on their platelet count because the disappearance of large VWF multimers is associated with transient or persistent thrombocytopenia [29]. Although the underlying mechanisms have yet to be fully elucidated, we can definitely conclude that there can be no consumption of large VWF multimers without thrombocytopenia, neither under basal conditions or after stress, nor during pregnancy, or after DDAVP infusion [30]. Under basal conditions, the type of VWF mutation influences a patient’s thrombocytopenia, since individuals carrying the p.V1316M mutation had the most pronounced and persistent drop in their platelet count, and they also had giant platelets. Patients carrying this particular mutation were first identified as having type Montreal thrombocytopenia, and it became clear only more recently that they have type 2B VWD with a pronounced thrombocytopenia [31].

After DDAVP administration, our type 2B VWD patients lacking in large multimers (but not those with a normal multimer pattern) developed a significant transient thrombocytopenia, which regressed within a few hours [14,19]. This finding clearly rules out the possibility of a true platelet consumption taking place because it would take considerably longer for the pre-infusion platelet count to be restored. This assumption is supported by the lack of any increase in circulating glycocalicin levels in such patients after DDAVP [32]. A similar picture was seen in pregnancy, a condition always associated with a progressive thrombocytopenia in females with type 2B VWD whose multimer pattern is abnormal [13, 16].

Other differences between our type 2B patients with normal or abnormal multimer patterns concern their platelet VWF content, which was always normal in cases lacking in large VWF multimers—a finding suggestive of a normal VWF synthesis. The picture was more heterogeneous in the patients with a normal multimer pattern, only some of whom had lower than normal levels of plasma and platelet VWF (in association with the p.R1379C and p.R1341W mutations).

The half-life of type 2B VWD patients’ VWF (measured after administering DDAVP) appeared to be reduced [30] and modulated by patients’ underlying mutations and multimer pattern, the shortest VWF half-life being seen in patients lacking in large VWF multimers. Consistently with a shorter VWF half-life, the VWFpp ratio also increased, particularly in patients lacking in large multimers.

The differences between our type 2B VWD patients with and without large VWF multimers also concerned their bleeding tendency, which was slightly more pronounced in the latter. Patients with a normal VWF multimer pattern had a more heterogeneous phenotype, however (some had a normal BS, while others had very high scores), whereas all but one of the patients lacking in large multimers had a similarly high BS. The tendency to bleed of our patients with an abnormal multimer pattern did not seem to be influenced by their platelet count, which revealed no statistical correlation with their BS. This finding is inconsistent with a previous report [13] that identified a reduced platelet count as a risk factor for bleeding in type 2B patients lacking in large VWF multimers.

Lastly, but no less importantly, a different approach is needed to the treatment of type 2B patients with and without large VWF multimers. DDAVP is appropriate, given its marked haemostatic efficacy, for type 2B VWD associated with a normal multimer pattern, and especially in patients with a normal platelet VWF content. Its use in patients with an abnormal multimer pattern seems to be controversial (mainly because they experience no post-DDAVP large multimer recovery), though it may be useful in the event of minor surgery, as in dental procedures [33]. Platelets are contraindicated for such patients because they could induce a further consumption of their residual large VWF multimers. Bearing these considerations in mind, failing to clearly distinguish between the two phenotypes of type 2B VWD may lead to the inappropriate treatment of these patients, especially at non-specialised centres or by non-expert physicians.

To conclude, type 2B VWD patients with normal and abnormal multimer patterns are similar in terms of their lower MADR, presence of SPA, increased VWF activation state, and shorter VWF half-life (all features associated with gain-of-function mutations in the A1 domain). They may differ, however, as regards the onset of thrombocytopenia, VWF synthesis, and bleeding tendency, so a different approach to their treatment is warranted. Table 3 shows the similarities and differences between type 2B with and without large VWF multimers.

**Table 3:** Synoptic representation of the differences and similarities between type 2B VWD patients with or without large VWF multimers.

2B VWD patients	Platelet count		^*^SPA	^°^MADR	VWF:Ag	^§^VWF:RCo ratio	^^^VWF:CB ratio	Platelet VWF:Ag	Large multimers	VWF activation state	VWF mutation in A1 domain	Bleeding tendency	Therapy		
	Pre	Post													
	DDAVP												DDAVP	Platelet	VWF/FVIII concentrates
Without multimers	↓/N	↓↓	Present	↓	↓	↓↓↓	↓↓↓	N	Absent	↑	Present	↑↑	No/Yes	No	Yes
With multimers	N	N	Present	↓	N/↓	N	N	N/↓	Present	↑	Present	↑	Yes	Yes	Yes

*Spontaneous platelet aggregation

°Minimal aggregating dose of ristocetin

§VWF:RCo/VWF:Ag

^VWF:CB/VWF:Ag.

N = normal; ↓ = reduced; ↑ = increased.

In the light of all the above-discussed issues, we would recommend grouping type 2B patients according to their VWF multimer pattern. As in type 2A VWD (in which patients are identified as type 2A-I when the defect is associated with a defective VWF synthesis and multimerisation, or as type 2A-II when their VWF defect is associated with an increased proteolysis of a normally-synthesised VWF), we suggest distinguishing between patients with type 2B-I, who are lacking in large multimers and type 2B-II, who have a normal VWF multimer pattern, with or without a normal VWF synthesis. This should enable a more precise classification of type 2B VWD patients, and ensure that they receive the most appropriate treatment.

## References

[1] Ruggeri ZM, Pareti FI, Mannucci PM, Ciavarella N, Zimmerman TS. Heightened interaction between platelets and factor VIII/von Willebrand factor in a new subtype of von Willebrand disease. N Engl J Med. 1980;302(19):1047–1051. doi: 10.1056/NEJM198005083021902 6767976 10.1056/NEJM198005083021902

[2] Ruggeri ZM. Type IIB von Willebrand disease: a paradox explains how von Willebrand factor works. J Thromb Haemost. 2004;2(1):2–6. 14717957 10.1111/j.1538-7836.2003.00523.x

[3] De Marco L, Girolami A, Zimmerman TS, Ruggeri ZM. Interaction of purified IIB von Willebrand factor with the platelet membrane glycoprotein Ib induces fibrinogen binding to the glycoprotein IIb-IIIa complex and initiates aggregation. Proc Natl Acad Sci U S A. 1985;82(21):7424–7428. 2932740 10.1073/pnas.82.21.7424 PMC391357

[4] Howard MA, Firkin BG. Ristocetin: a new tool in the investigation of platelet aggregation. Thromb Diath Haemorrh. 1971;26(2):362–369. 5316292

[5] Howard MA, Sawers RJ, Firkin BG. Ristocetin. A means of differentiating von Willebrand's disease into two groups. Blood. 1973;41(5):687–690. 4540393

[6] De Marco L, Mazzucato M, Del Ben MG, Budde U, Federici AB, Girolami A, et al Type IIB von Willebrand factor with normal sialic acid content induces platelet aggregation in the absence of ristocetin. Role of platelet activation, fibrinogen, and two distinct membrane receptors. J Clin Invest. 1987;80(2):475–482. doi: 10.1172/JCI113095 3038958 10.1172/JCI113095 PMC442260

[7] Randi AM, Rabinowitz I, Mancuso DJ, Mannucci PM, Sadler JE. Molecular basis of von Willebrand disease type IIB: candidate mutations cluster in one disulfide loop between proposed platelet glycoprotein Ib binding sequences. J Clin Invest. 1991;87(4):1220–1226. doi: 10.1172/JCI115122 2010538 10.1172/JCI115122 PMC295140

[8] Cooney KA, Nichols WC, Bruck ME, Bahou WF, Shapiro AD, Bowie EJ, et al The molecular defect in type IIB von Willebrand disease: identification of four potential missense mutations within the putative GpIb binding domain. J Clin Invest. 1991;87(4):1227–1233. doi: 10.1172/JCI115123 1672694 10.1172/JCI115123 PMC295141

[9] Ruggeri ZM, Zimmerman TS. The complex multimeric composition of Factor VIII/von Willebrand factor. Blood. 1981;57(6):1140–1143. 6784794

[10] Hoyer LW, Shainoff JR. Factor VIII-related protein circulates in normal human plasma as high molecular weight multimers. Blood. 1980;55(6):1056–1059. 6769518

[11] Gralnick HR, Williams SB, Morisato DK. Effect of multimeric structure of the factor VIII/von Willebrand factor protein on binding platelets. Blood. 1981;58(2):387–397. 6788111

[12] Rayes J, Hommais A, Legendre P, Tout H, Veyradier A, Obert B, et al Effect of von Willebrand disease type 2B and type 2M mutations on the susceptibility of von Willebrand factor to ADAMTS-13. J Thromb Haemost. 2007;5(2):321–328. doi: 10.1111/j.1538-7836.2007.02296.x 17087728 10.1111/j.1538-7836.2007.02296.x

[13] Federici AB, Mannucci PM, Castaman G, Baronciani L, Bucciarelli P, Canciani MT, et al Clinical and molecular predictors of thrombocytopenia and risk of bleeding in patients with von Willebrand disease type 2B: a cohort study of 67 patients. Blood. 2009;113(3):526–534. doi: 10.1182/blood-2008-04-152280 18805962 10.1182/blood-2008-04-152280

[14] Casonato A, Fabris F, Girolami A. Platelet aggregation and pseudothrombocytopenia induced by 1-desamino-8-D-arginine vasopressin (DDAVP) in type IIB von Willebrand's disease patient. Eur J Haematol. 1990;45(1):36–42. 2116314 10.1111/j.1600-0609.1990.tb00412.x

[15] Rick ME, Williams SB, Sacher RA, McKeown LP. Thrombocytopenia associated with pregnancy in a patient with type IIB von Willebrand disease. Blood. 1987;69(3):786–789. 3028536

[16] Casonato A, Sartori MT, Bertomoro A, Fede T, Vasoin F, Girolami A. Pregnancy-induced worsening of thrombocytopenia in a patient with type IIB von Willebrand's disease. Blood Coagul Fibrinolysis. 1991;2(1):33–40. 1772996 10.1097/00001721-199102000-00005

[17] Hultin MB, Sussman II. Postoperative thrombocytopenia in type IIB von Willebrand disease. Am J Hematol. 1990;33(1):64–68. 2293764 10.1002/ajh.2830330113

[18] Holmberg L, Nilsson IM, Borge L, Gunnarsson M, Sjorin E. Platelet aggregation induced by 1-desamino-8-D-arginine vasopressin (DDAVP) in type IIB von Willebrand disease. N Engl J Med. 1983;309(14):816–821. doi: 10.1056/NEJM198310063091402 6412139 10.1056/NEJM198310063091402

[19] Casonato A, Sartori MT, de Marco L, Girolami A. 1-Desamino-8-D-arginine vasopressin (DDAVP) infusion in type IIB von Willebrand's disease: shortening of bleeding time and induction of a variable pseudothrombocytopenia. Thromb Haemost. 1990;64(1):117–120. 2274916

[20] Weiss HJ, Sussman II. A new von Willebrand variant (type I, New York): increased ristocetin-induced platelet aggregation and plasma von Willebrand factor containing the full range of multimers. Blood. 1986;68(1):149–156. 3487353

[21] Holmberg L, Dent JA, Schneppenheim R, Budde U, Ware J, Ruggeri ZM. von Willebrand factor mutation enhancing interaction with platelets in patients with normal multimeric structure. J Clin Invest. 1993;91(5):2169–2177. doi: 10.1172/JCI116443 8486782 10.1172/JCI116443 PMC288219

[22] Casonato A, De Marco L, Mazzucato M, De Angelis V, De Roia D, Fabris F, et al A new congenital platelet abnormality characterized by spontaneous platelet aggregation, enhanced von Willebrand factor platelet interaction and the presence of all von Willebrand factor multimers in plasma. Blood. 1989;74(6):2028–2033. 2804346

[23] Casonato A, Pontara E, Bertomoro A, Zucchetto S, Zerbinati P, Girolami A. Abnormal collagen binding activity of 2A von Willebrand factor: evidence that the defect depends only on the lack of large multimers. J Lab Clin Med. 1997;129(2):251–259. 9016863 10.1016/s0022-2143(97)90147-5

[24] Gallinaro L, Cattini MG, Sztukowska M, Padrini R, Sartorello F, Pontara E, et al A shorter von Willebrand factor survival in O blood group subjects explains how ABO determinants influence plasma von Willebrand factor. Blood. 2008;111(7):3540–3545. doi: 10.1182/blood-2007-11-122945 18245665 10.1182/blood-2007-11-122945

[25] Mancuso DJ, Tuley EA, Westfield LA, Worrall NK, Shelton-Inloes BB, Sorace JM, et al Structure of the gene for human von Willebrand factor. J Biol Chem. 1989;264(33):19514–19527. 2584182

[26] Casonato A, Sartorello F, Pontara E, Gallinaro L, Bertomoro A, Cattini MG, et al A novel von Willebrand factor mutation (I1372S) associated with type 2B-like von Willebrand disease: an elusive phenotype and a difficult diagnosis. Thromb Haemost. 2007;98(6):1182–1187. 18064311 10.1160/th07-05-0347

[27] Casonato A, Pontara E, Morpurgo M, Sartorello F, De Groot PG, Cattini MG, et al Higher and lower active circulating VWF levels: different facets of von Willebrand disease. Br J Haematol. 2015;171(5):845–853. doi: 10.1111/bjh.13785 26456374 10.1111/bjh.13785

[28] Hulstein JJ, de Groot PG, Silence K, Veyradier A, Fijnheer R, Lenting PJ. A novel nanobody that detects the gain-of-function phenotype of von Willebrand factor in ADAMTS 13 deficiency and von Willebrand disease type 2B. Blood. 2005;106(9):3035–3042. doi: 10.1182/blood-2005-03-1153 16014562 10.1182/blood-2005-03-1153

[29] Ozeki M, Kunishima S, Kasahara K, Funato M, Teramoto T, Kaneko H, et al A family having type 2B von Willebrand disease with an R1306W mutation: severe thrombocytopenia leads to the normalization of high molecular weight multimers. Thromb Res. 2010;125(2):e17–22. doi: 10.1016/j.thromres.2009.08.012 19740526 10.1016/j.thromres.2009.08.012

[30] Casonato A, Gallinaro L, Cattini MG, Pontara E, Padrini R, Bertomoro A, et al Reduced survival of type 2B von Willebrand factor, irrespective of large multimer representation or thrombocytopenia. Haematologica. 2010;95(8):1366–1372. doi: 10.3324/haematol.2009.019927 20305138 10.3324/haematol.2009.019927 PMC2913086

[31] Jackson SC, Sinclair GD, Cloutier S, Duan Z, Rand ML, Poon MC. The Montreal platelet syndrome kindred has type 2B von Willebrand disease with the VWF V1316M mutation. Blood. 2009;113(14):3348–3351. doi: 10.1182/blood-2008-06-165233 19060241 10.1182/blood-2008-06-165233

[32] Casonato A, Steffan A, Pontara E, Zucchetto A, Rossi C, De Marco L, et al Post-DDAVP thrombocytopenia in type 2B von Willebrand disease is not associated with platelet consumption: failure to demonstrate glycocalicin increase or platelet activation. Thromb Haemost. 1999;81(2):224–228. 10063996

[33] Casonato A, Pontara E, Dannhaeuser D, Bertomoro A, Sartori MT, Zerbinati P, et al Re-evaluation of the therapeutic efficacy of DDAVP in type IIB von Willebrand's disease. Blood Coagul Fibrinolysis. 1994;5(6):959–964. 7893933 10.1097/00001721-199412000-00013

